# Mechanical Performance of Basalt Fiber-Reinforced Fully Recycled Concrete Using Triple-Modified Recycled Aggregates

**DOI:** 10.3390/ma19061190

**Published:** 2026-03-18

**Authors:** Xinzhong Wang, Biao Zhou, Weidong Cheng, Yuwen Sun, Eguo Xiao, Zhengyi He

**Affiliations:** College of Civil Engineering, Hunan City University, Yiyang 413000, China

**Keywords:** multiple modification, interfacial transition zone, basalt fiber, fully recycled concrete, synergistic reinforcement mechanism

## Abstract

Aiming at the critical problem that recycled concrete aggregate (RCA) has more cracks and severe defects on its surface than natural aggregate, resulting in an excessively weak interfacial transition zone (ITZ) between RCA and cement paste, this paper proposes a triple synergistic modification method combining calcium ion accelerating solution treatment, dopamine polymerization treatment and nanofiber reinforcement to improve the properties of recycled aggregate. Through in-depth research on the mechanical properties of basalt fiber-reinforced fully recycled concrete after triple modification, it is found that the triple modification technology can significantly optimize the structure of the recycled aggregate-cement paste ITZ. The 28-day compressive strength of the fully recycled concrete is increased by 56% (reaching 27.7 MPa), and the splitting tensile strength is improved by 129% (reaching 5.32 MPa). Microscopic analysis shows that the modified system realizes gradient strengthening of the ITZ structure through the synergistic mechanism of “pore filling, chemical bonding and fiber bridging”. This research provides a new idea for the high-performance modification of fully recycled concrete, and has important significance for promoting the sustainable development of the construction industry.

## 1. Introduction

The resource utilization of construction waste is an important approach to promoting the sustainable development of civil engineering. According to statistics, China generates more than 3 billion tons of construction waste annually, among which waste concrete accounts for over 40% [1]. Processing waste concrete into RCA to replace natural aggregate can not only alleviate the shortage of sand and gravel resources, but also reduce carbon dioxide emissions by approximately 15% [2,3,4]. However, the old mortar adhering to the surface of RCA results in its porosity (7–12%) and water absorption rate (3–8%) being significantly higher than those of natural aggregate, and there are a large number of microcracks in the ITZ, which seriously restricts the mechanical properties and engineering application of recycled concrete [5,6,7].

In recent years, significant progress has been made in research on modification techniques for RCA. In terms of physical modification, mechanical grinding can reduce the water absorption of aggregates by 18–25%, but it may exacerbate internal micro-damage and indirectly affect the long-term stability of recycled concrete [8,9,10]. For biological modification, microbial mineralization (e.g., MICP) is utilized to deposit calcium carbonate, which can increase the compressive strength of concrete by 12–15%; however, the treatment period typically lasts as long as 14–28 days, failing to meet the efficiency requirements for large-scale engineering applications [11,12,13]. Among chemical modification methods, treatment with nano-SiO_2_ solution can improve the microhardness of the ITZ by approximately 40%, while PVA impregnation can significantly reduce the chloride ion diffusion coefficient. Additionally, acid treatment combined with mechanical grinding has been proven effective in improving the interfacial properties of aggregates [14,15,16,17,18]. Nevertheless, existing methods mostly focus on optimizing single properties, making it difficult to systematically strengthen the ITZ structure and synergistically enhance the comprehensive performance of recycled concrete.

Existing single modification methods typically only optimize a specific weak link in recycled concrete, making it difficult to comprehensively improve its multi-scale structural defects. Moreover, traditional multi-modification approaches often employ a simple superposition strategy, implementing different modification methods sequentially and independently, lacking chemical coupling and synergistic design among various modification elements, which hinders the synergistic improvement of mechanical properties and durability of recycled concrete. The triple synergistic modification strategy combining calcium ion treatment, dopamine coating, and nanofibers, along with its multi-scale synergistic mechanism spanning chemical activation, interfacial strengthening, and microstructural regulation, will provide new theoretical insights for interfacial engineering in recycled concrete. Based on this, this study proposes a triple synergistic modification strategy: (1) using calcium ion promoting solution to facilitate C-S-H gel generation through alkaline activation, thereby filling aggregate pores; (2) utilizing the catechol groups of polydopamine to form coordination bonds with cement hydration products, thus enhancing interfacial chemical bonding; (3) introducing nanofibers to construct a three-dimensional network structure in the ITZ region to inhibit micro-crack propagation. Through multi-scale characterization and mechanical property testing, the influence patterns of the modification system on the performance of basalt fiber fully recycled concrete will be systematically investigated, providing theoretical support and technical pathways for developing high-performance recycled concrete.

## 2. Materials and Methods

### 2.1. Experimental Materials

**Figure 1:**
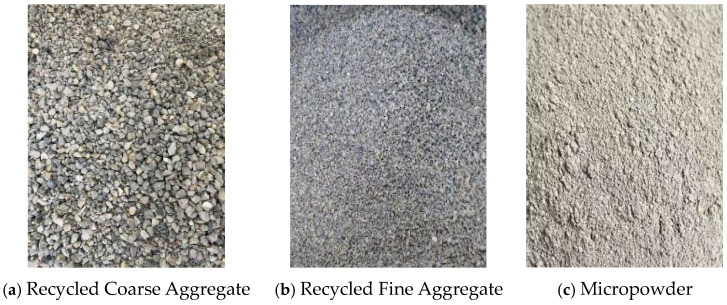
Coarse and fine aggregates and micro-powder materials.

**Figure 2:**
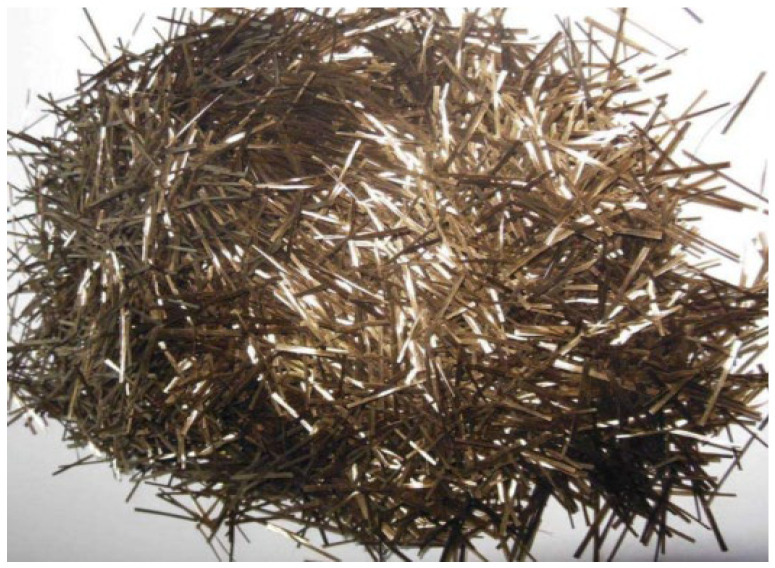
18 mm Basalt Fiber.

**Figure 3:**
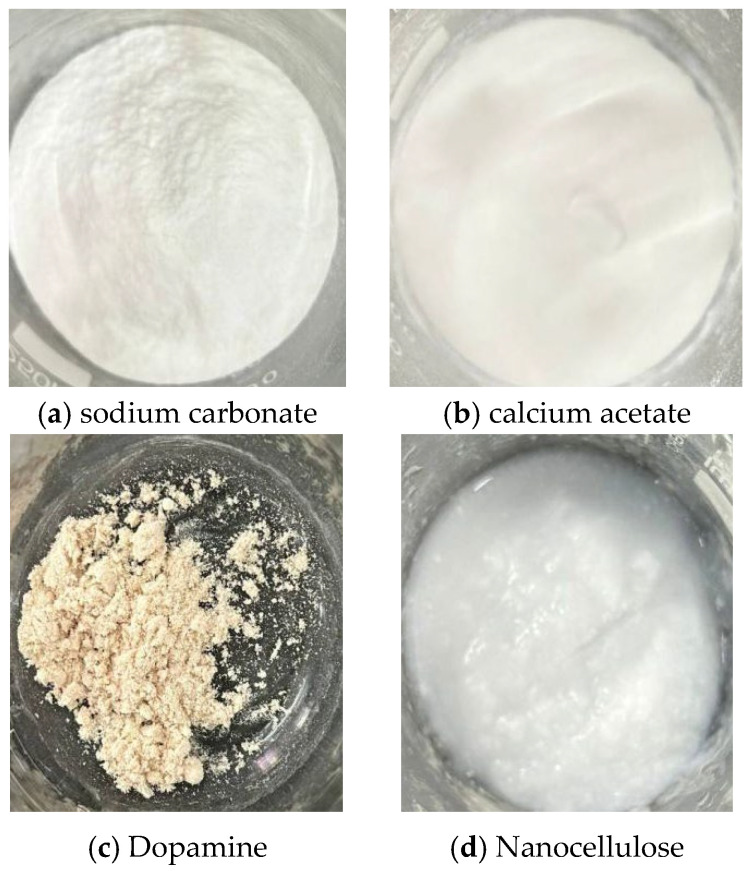
Modification Materials.

Portland cement (P.O 42.5) was selected as the cementitious material. The coarse aggregate, fine aggregate, and micro-powder materials are illustrated in Figure 1, while the mix proportion data are presented in Table 1. The basalt fibers used were chopped basalt fibers produced by Zhejiang Hengdian Shijin Basalt Fiber Co., Ltd, China, with the specific parameters as follows: fiber diameter of 17 μm, fiber density of 26 kg/m^3^, tensile strength of 3000 MPa, elastic modulus of 90 GPa, and fiber length of 18 mm, as shown in Figure 2. The modification materials included a calcium ion accelerating solution (prepared from sodium carbonate and calcium acetate), a dopamine solution, and a nanofiber dispersion, which are depicted in Figure 3.

### 2.2. Aggregate Modification

**Figure 4:**
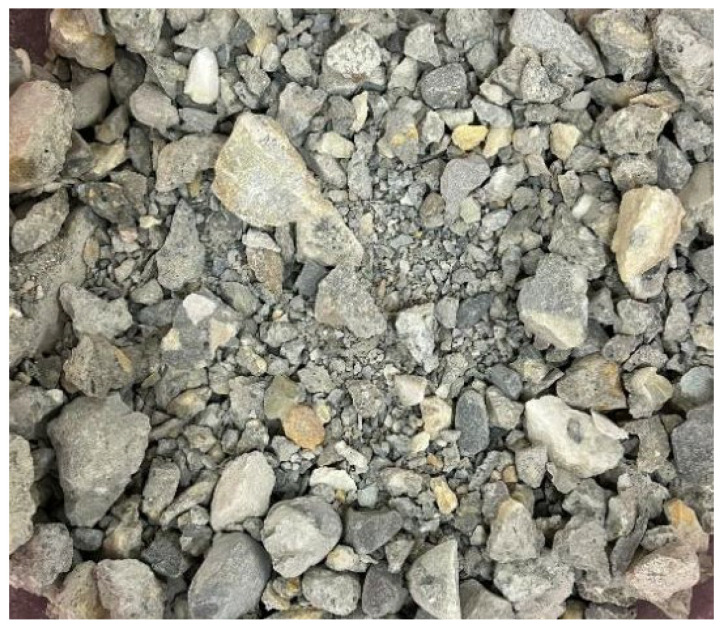
Raw Recycled Aggregate.

**Figure 5:**
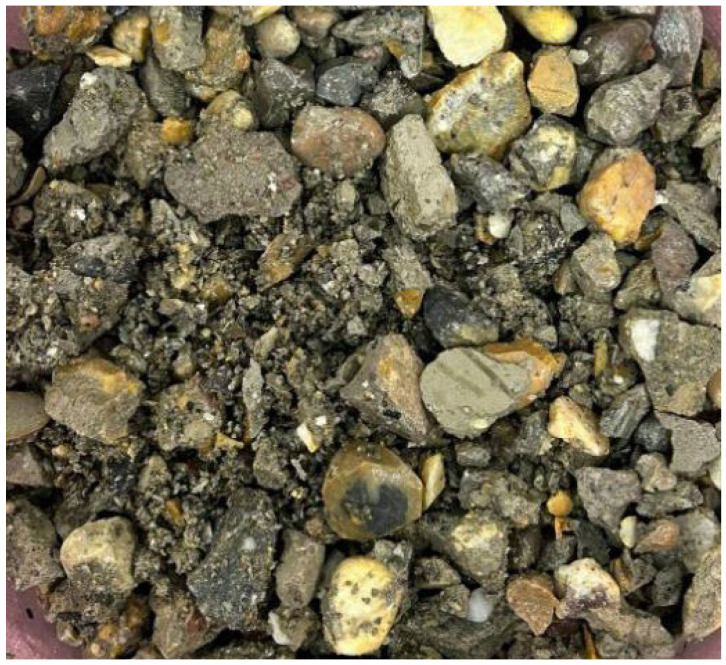
Modification with Calcium Ion Accelerating Solution.

**Figure 6:**
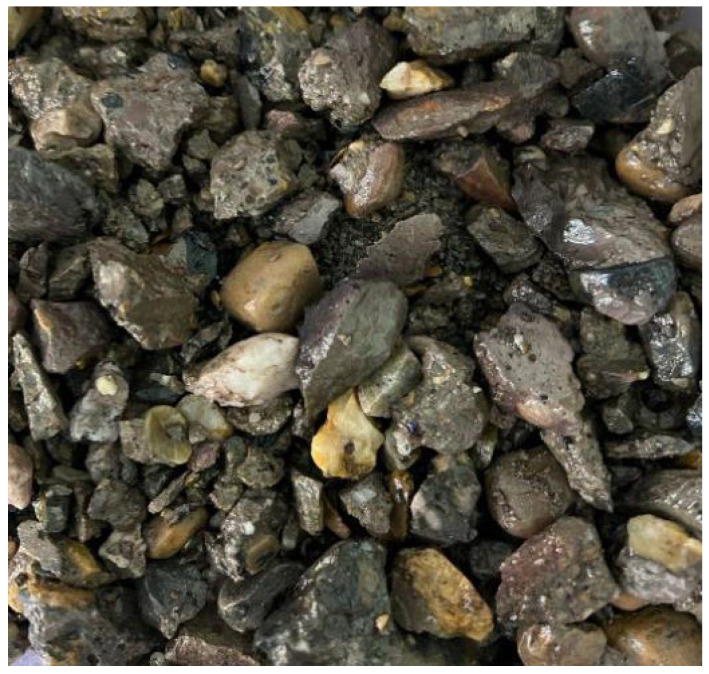
Modification with Dopamine Solution.

**Figure 7:**
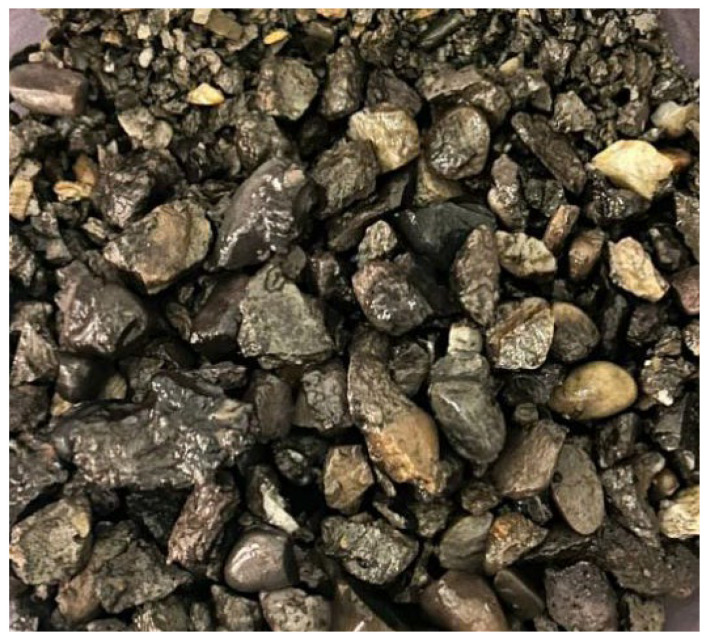
Modification with Nanocellulose.

The recycled aggregate (as shown in Figure 4) was successively treated with calcium ion-containing accelerating solution, dopamine polymer, and nanofiber modification. The recycled aggregate was immersed in the calcium ion-containing accelerating solution, followed by the addition of sodium carbonate solution. After a reaction for 30–60 min, the aggregate was dried to complete the treatment with calcium ion-containing accelerating solution (as shown in Figure 5). The calcium ion-containing accelerating solution was calcium acetate, with a calcium ion concentration ranging from 0.0125 M to 0.125 M. The carbonate solution was sodium carbonate, where the molar ratio of carbonate ion concentration to calcium ion concentration in the carbonate solution was 0.9–1.1:1. Subsequently, the recycled aggregate was immersed in a dopamine solution with a concentration of 1–10 mg/mL. The pH value of the dopamine solution was then adjusted to above 8.5, and the reaction was carried out for 12 h. After drying, the dopamine polymerization treatment was completed (as shown in Figure 6). Finally, the recycled aggregate treated by dopamine polymerization was immersed in a nanofiber dispersion containing 0.01–0.1% (by mass) of nanofibers and 0.1–1% (by mass) of anionic surfactant. After standing for 24 h and drying, the nanofiber-modified recycled aggregate was obtained (as shown in Figure 7).

### 2.3. Mechanical Property Test

**Figure 8:**
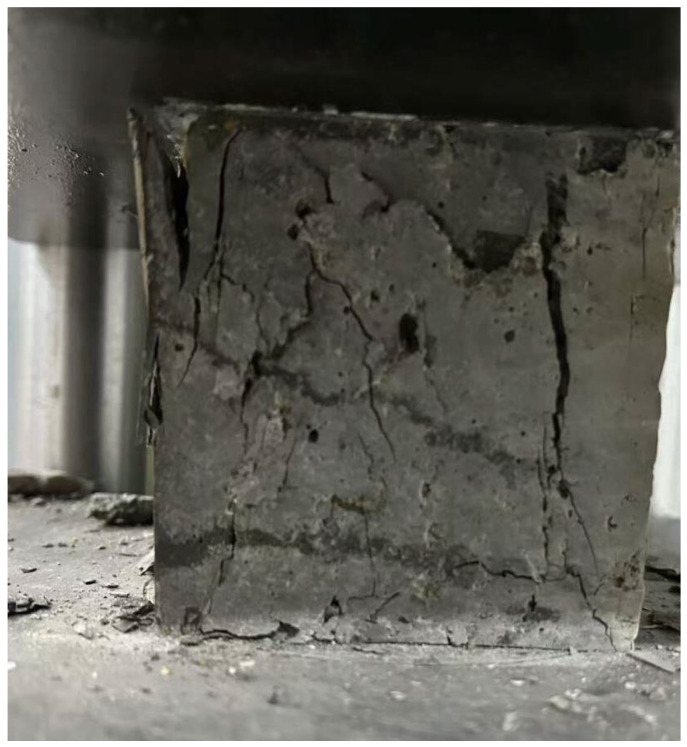
Compressive Strength Test.

**Figure 9:**
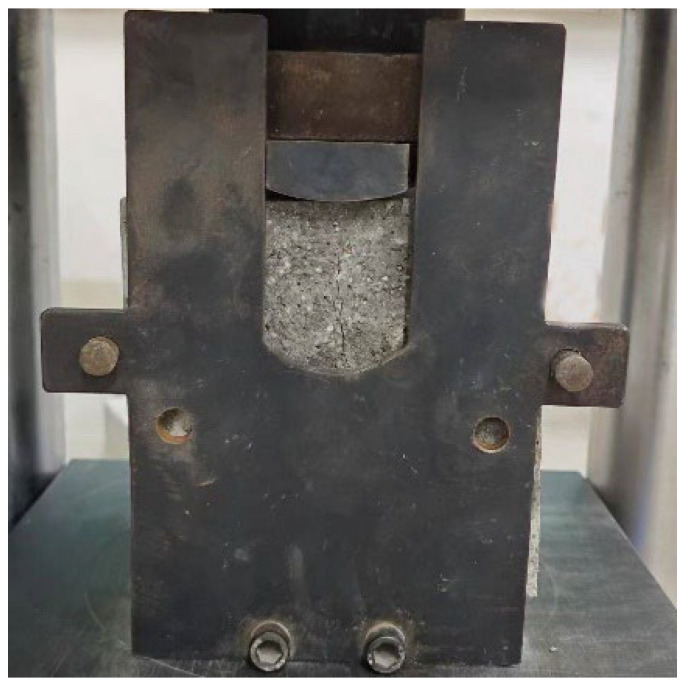
Tensile Strength Test.

According to the Standard for Test Methods of Physical and Mechanical Properties of Concrete (GB/T 50081-2019) [19], a total of 72 concrete specimens were prepared, consisting of 12 groups with 6 specimens per group. The concrete mixture was evenly poured into 150 mm × 150 mm × 150 mm cubic molds in two layers. Each layer was manually tamped from the periphery toward the center. After tamping, the molds were gently tapped around with a rubber mallet until voids left by the tamping rod disappeared. Excess concrete was then scraped off from the top of the molds. When the concrete approached the initial setting stage, the surface was smoothed with a scraper and covered with a plastic film. The specimens were left to stand for 24 h at a temperature of 20 ± 5 °C before demolding and numbering. Subsequently, all specimens were cured for 28 days in an environment maintained at 20 ± 2 °C with a relative humidity of over 95%. After curing, compressive strength tests (as shown in Figure 8) and tensile strength tests (as shown in Figure 9) were conducted.

## 3. Results

### 3.1. Test Results

The test results are presented in Table 2. Compared with the unmodified group, the specimens subjected to triple modification exhibited a 56% increase in compressive strength and a 129% increase in tensile strength. It can be concluded that triple modification significantly enhances the compressive and tensile strengths of basalt fiber-reinforced fully recycled concrete specimens. In comparison with Example 1, Comparative Example 1 omitted the nanofiber modification step and instead incorporated nanofibers directly into the mixture as a filler, resulting in slightly inferior performance to Example 1. Comparative Example 2 lacked the calcium ion accelerating solution treatment step, which led to a further decline in performance. Comparative Example 3 excluded the dopamine polymerization treatment step, causing reduced performance. Comparative Example 4 also omitted the nanofiber modification step, resulting in performance slightly lower than that of Example 1. Comparative Example 5 used a higher concentration of dopamine solution than Example 1, which formed a thick polydopamine film on the surface of recycled aggregates and compromised the modification effect, thus leading to a slight decrease in performance. In Comparative Example 6, the mass percentage of nanofibers was excessively high, resulting in poor dispersibility in the dispersion; the agglomeration of nanofibers impaired the modification effect, so its performance was similar to that of Comparative Example 1. Comparative Example 7 involved recycled aggregates without any modification treatment, achieving a compressive strength of only 49.5 MPa and a tensile strength of 4.13 MPa, which reflects the inherent defects of recycled aggregates.

**Figure 10:**
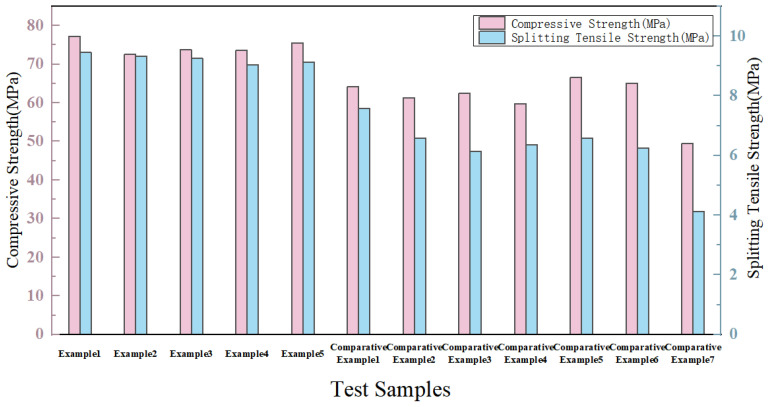
Bar Chart of Test Data.

**Figure 11:**
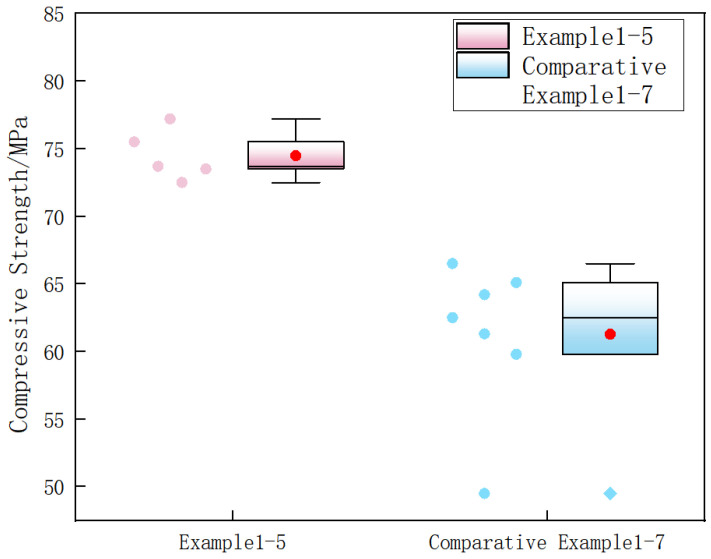
Box Plot of Test Data.

**Figure 12:**
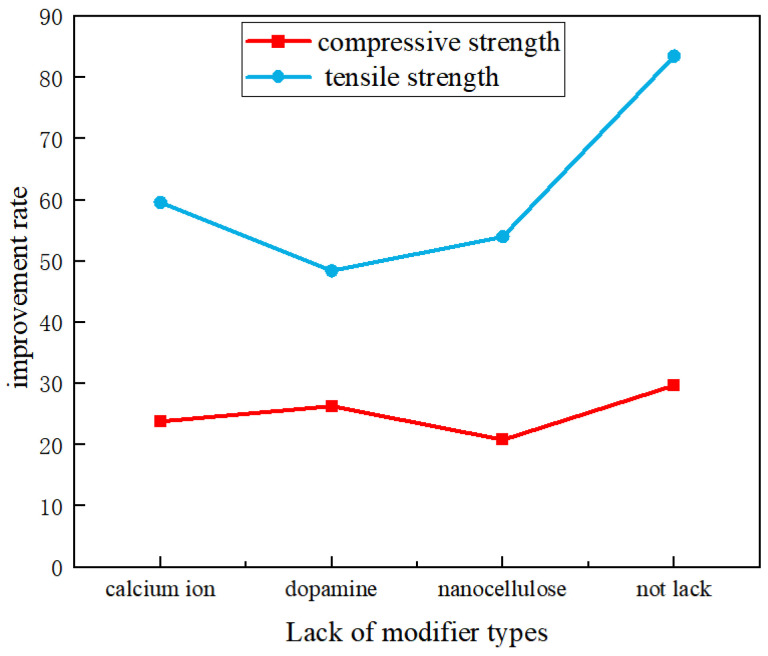
Strength Improvement Rate of Double-modified Concrete.

In addition, all the modified specimens achieved a compressive strength of over 70 MPa and a tensile strength of over 9 MPa, which were significantly higher than those of the comparative examples. Moreover, the effect of triple modification was obviously superior to that of double modification. Summarizing the test data in Table 2, it can be concluded that among the three modifiers, the absence of nanofibers exerted the most significant impact on the mechanical properties of the specimens, followed by the dopamine solution, while the calcium ion accelerating solution had a relatively minor effect. Based on the data in Table 2, the bar chart, box plot, and line chart of the strength improvement rate of double-modified concrete were plotted, as shown in Figure 10, Figure 11 and Figure 12.

### 3.2. Result Analysis

Based on the analysis of Figure 10 and Figure 11, in conjunction with the data in Table 2, it can be observed that the compressive strengths of Examples 1–5 all exceeded 70 MPa. Compared to the unmodified specimens in the comparative examples, the compressive strength improvement rate ranged from 46.5% to 56%, indicating that the triple modification had a significant enhancement effect on the compressive strength of basalt fiber-reinforced fully recycled concrete. The compressive strengths of all specimens in Comparative Examples 1–7 were lower than those in Examples 1–5. It can therefore be concluded that excessively high modifier concentrations also diminish the enhancement effect on compressive strength, and that double modification was significantly less effective than triple modification.

As shown in Figure 12, the omission of any one of the three modification steps—calcium ion treatment, dopamine coating, or nanocellulose incorporation—leads to a reduction in both compressive strength and tensile strength. This reduction has a relatively minor impact on compressive strength but a more significant effect on tensile strength. Compared with tensile strength, single modifications have a very limited effect on improving compressive strength. Compared with Example 1, the compressive strengths of Comparative Examples 2–4 (each lacking one modifier) decreased by 21.2–28.1%, while the tensile strengths dropped by 39.4–52.1%. This confirms that double modification is less effective than triple modification in enhancing concrete performance. From the perspective of compressive strength, among the three modifiers, the absence of nanofibers had the greatest negative impact, followed by the calcium ion solution, and finally the dopamine solution. In terms of tensile strength, the omission of dopamine modification caused the most significant loss, followed by nanofibers, and then the calcium ion solution.

As shown in Table 2, when the dopamine concentration was excessively high (Comparative Example 5, 20 mg/mL), the compressive strength decreased to 66.5 MPa and the tensile strength dropped to 6.57 MPa, suggesting that an overly high dopamine concentration may lead to strength reduction. When the nanofiber content was too high (Comparative Example 6, 0.2%), the compressive strength decreased to 65.1 MPa and the tensile strength fell to 6.25 MPa, which may be attributed to fiber agglomeration causing stress concentration, thereby degrading the mechanical properties of the recycled concrete. The compressive strength of Example 2 (calcium ion concentration: 0.125 M) was 72.5 MPa, lower than that of Example 1 (0.1 M) at 77.2 MPa, indicating that an excessively high calcium ion concentration may lead to crystal coarsening, weaken the interface performance, and consequently impair the mechanical properties of the recycled concrete.

### 3.3. Mechanism Analysis

**Figure 13:**
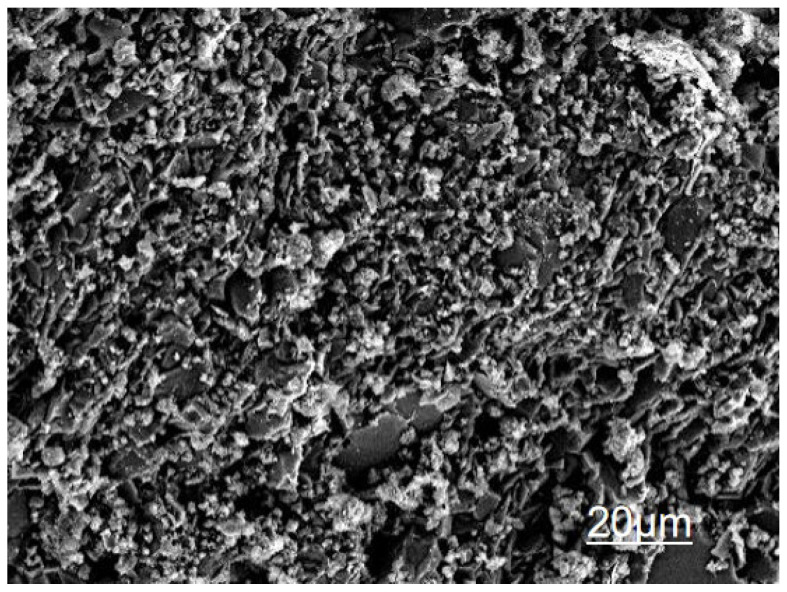
Raw Recycled Aggregate.

**Figure 14:**
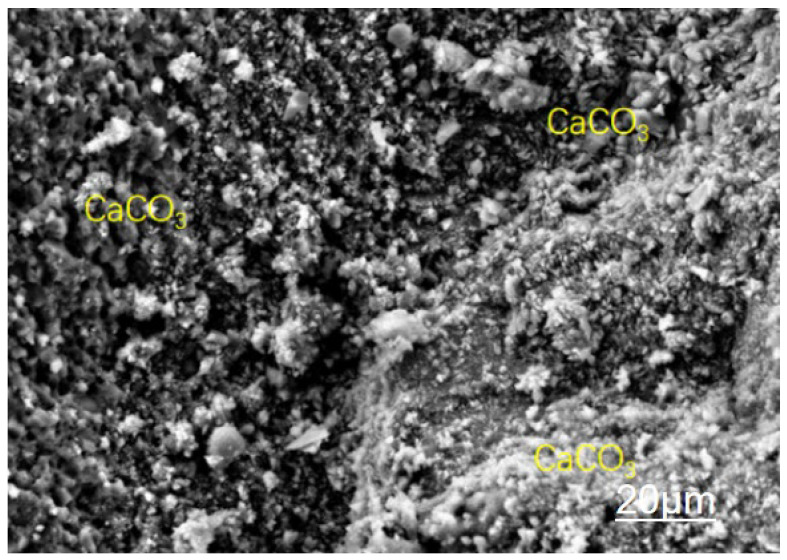
Modification with Calcium Ion Accelerating Solution.

**Figure 15:**
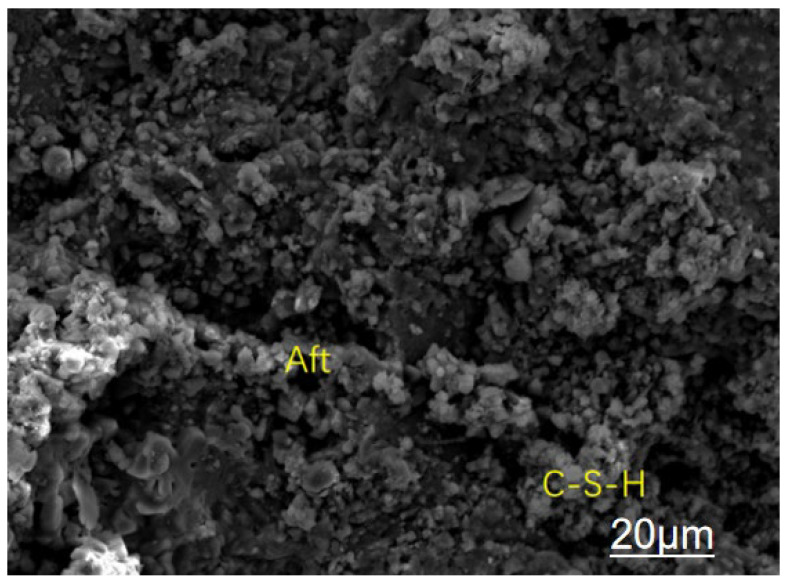
Modification with Dopamine Solution.

**Figure 16:**
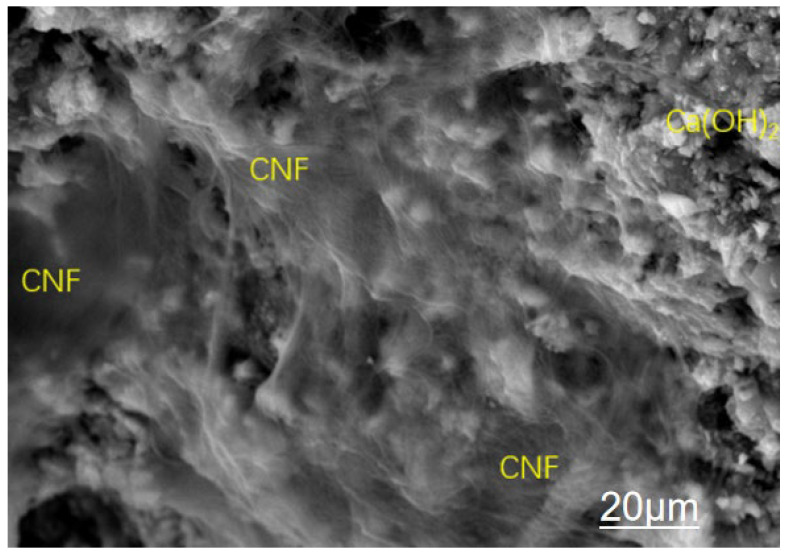
Modification with Nanocellulose.

The triple modification technology enhances the mechanical properties of recycled concrete across multiple scales, from the micro to the macro level, through the synergistic effects of a calcium ion accelerating solution, a dopamine solution, and nanofibers. Figure 13, Figure 14, Figure 15 and Figure 16 present SEM images of unmodified aggregates and aggregates modified with each of the three reagents, respectively.

**Figure 17:**
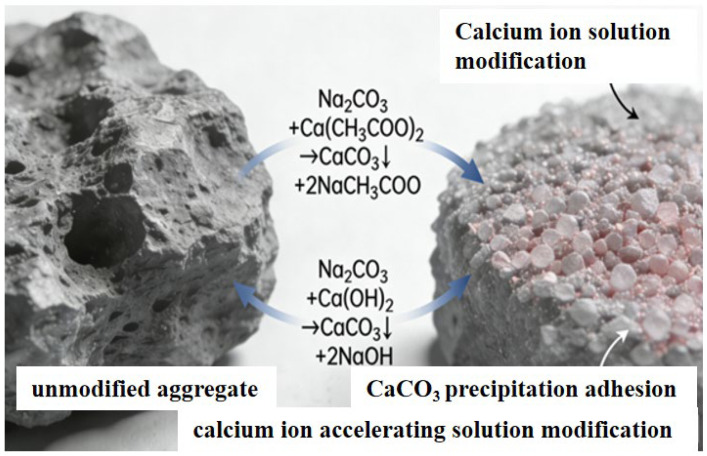
Schematic Diagram of Modification with Calcium Ion Accelerating Solution.

A combined analysis of Figure 14 and Figure 17 reveals that after modification treatment with calcium ion solution, the amount of hydration products attached to the surface of the recycled aggregates increases significantly, and the compactness of the overall structure is notably enhanced. This phenomenon can be explained by the reaction process depicted in the figures from two perspectives: first, sodium carbonate undergoes a double decomposition reaction with calcium acetate in aqueous solution to produce calcium carbonate (CaCO_3_) precipitate and soluble sodium acetate (NaCH_3_COO). The reaction equation is as follows:

$$Na_{2}CO_{3}+Ca{\left(CH_{3}COO\right)}_{2}\to CaCO_{3}↓+2NaCH_{3}COO$$

The generated calcium carbonate is dispersed in the solution in the form of micron-sized particles, which gradually deposit and adhere to the aggregate surface, forming a dense coating layer. Second, the sodium carbonate solution itself is strongly alkaline (pH > 10) and can dissolve the free calcium hydroxide (Ca(OH)_2_) present on the surface of the recycled aggregate, after which a subsequent reaction occurs:

$$Na_{2}CO_{3}+Ca{\left(OH\right)}_{2}\to CaCO_{3}↓+2NaOH$$

This dual reaction mechanism not only increases the amount of surface deposits but also significantly enhances the compactness and structural stability of the aggregate interface by filling surface pores and covering weak areas. This explains the improved surface morphology and enhanced performance of the modified aggregates observed in the figures.

**Figure 18:**
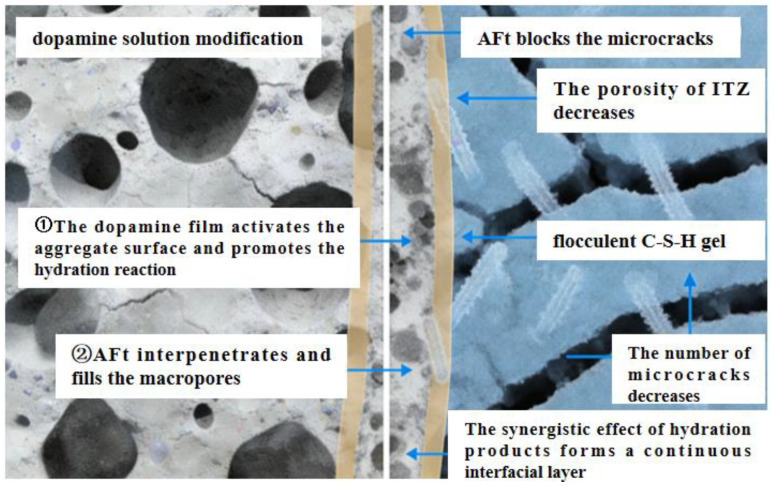
Schematic Diagram of Modification with Dopamine Solution.

An analysis of Figure 15 shows that after modification with dopamine solution, a large number of hydration products such as ettringite and C-S-H gel are formed in the pores between recycled aggregates and cement paste, effectively filling the pores at the interfacial transition zone (ITZ) and reducing the porosity of the ITZ. Combined with Figure 18, the surface of unmodified recycled aggregates has obvious large pores (as shown on the left side of Figure 18), and the interfacial transition zone (ITZ) between the aggregates and cement paste has high porosity and a loose structure. In contrast, after modification with dopamine solution, hydration products—including ettringite crystals and C-S-H gel—are generated in the pores between recycled aggregates and cement paste. These products can effectively fill the voids in the ITZ and significantly reduce the porosity of this zone.

From the perspective of microscopic mechanisms, the old mortar originally attached to the surface of recycled aggregates tends to form a porous and weak interface. However, dopamine modification promotes the hydration reaction, thereby enabling products such as ettringite and C-S-H gel to “fill” the pores and microcracks in the ITZ. This process is equivalent to strengthening the bonding interface between aggregates and cement paste. This change directly reduces the stress concentration points at the ITZ, thus not only lowering the risk of microcrack initiation and propagation, but also improving the compactness of the interface. Ultimately, this improvement is reflected in the enhanced mechanical properties (e.g., compressive strength and flexural strength) of recycled concrete. Meanwhile, due to the reduced pore connectivity, its durability performances such as carbonation resistance and impermeability are also improved.

This indicates that soaking recycled aggregates in dopamine hydrochloride solution can reduce the microcracks in the interfacial transition zone between recycled aggregates and cement paste, thereby improving the mechanical properties and durability of recycled concrete.

**Figure 19:**
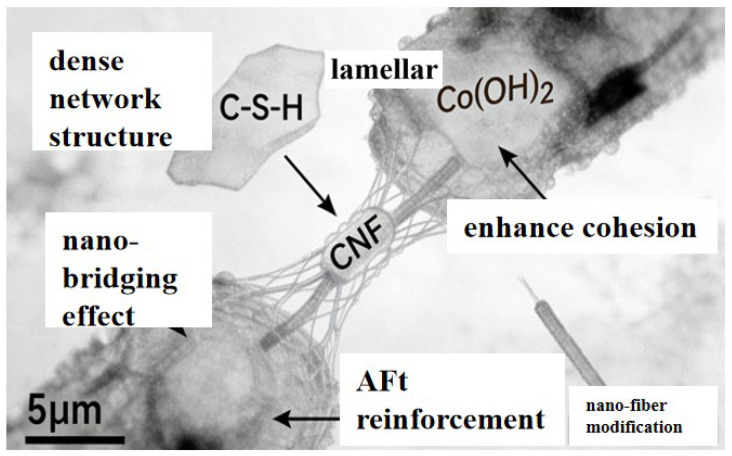
Schematic Diagram of Nanofiber Modification.

It can be seen from the combined analysis of Figure 16 and Figure 19 that cellulose nanofibers (CNF) are substances with a smaller size than cement hydration products, namely calcium silicate hydrate, calcium hydroxide and ettringite (AFt). They tend to form a dense network structure, tightly wrapping fibrous C-S-H gel, flaky Ca(OH)_2_ crystals, and needle-shaped ettringite (AFt). It is evident that, owing to their ultra-small size and ability to form a dense network with cement hydration products, CNF can enhance the compactness of the new interfacial transition zone (ITZ) in recycled concrete. Furthermore, the nanobridging effect of CNF establishes connections between cement hydration products, strengthening the cohesion within the cement matrix, delaying its fracture, and consequently improving the bonding performance between recycled aggregates and the cement matrix.

**Figure 20:**
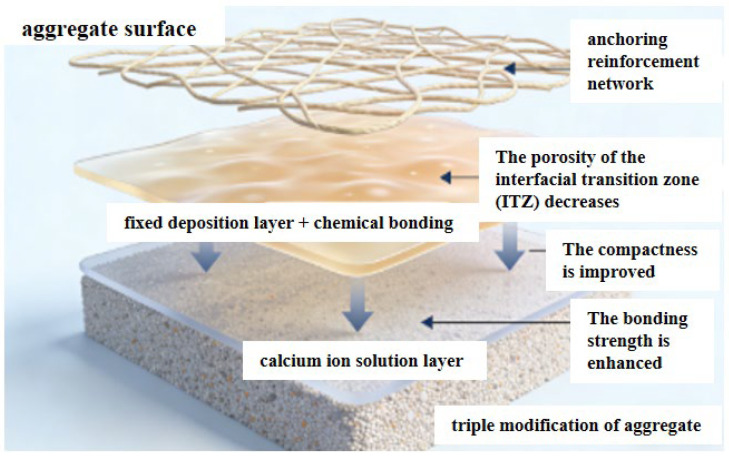
Schematic Diagram of Triple Modification of Aggregates.

The synergistic effect of the triple modification is manifested through multi-level enhancement mechanisms (as shown in Figure 20). First, calcium ion deposition provides active sites for the polymerization of dopamine, facilitating its efficient adhesion. Subsequently, the formed dopamine film can not only immobilize the calcium ion deposition layer but also form chemical bonds with nanofibers, further strengthening the interfacial connection. Meanwhile, nanofibers achieve anchoring via the calcium ion deposition layer, jointly constructing a stable three-dimensional reinforcing network.

This multi-level synergistic effect significantly reduces the porosity of the interfacial transition zone (ITZ) of concrete, enhances the compactness and interfacial bonding strength of this zone, and thus comprehensively improves the overall mechanical properties of concrete. The modified recycled aggregates exhibit higher surface strength and better interfacial bonding capacity, which helps to alleviate the weak points in the interfacial transition zone. On this basis, their synergistic effect with basalt fibers further improves the crack resistance of concrete, enabling the material to exhibit better durability and structural integrity under load.

## 4. Conclusions

In this study, a triple synergistic modification method involving calcium ion accelerating solution treatment, dopamine polymerization reaction, and nanofiber reinforcement was adopted to optimize the structure of the interfacial transition zone (ITZ) between recycled aggregates and cement paste, thereby significantly enhancing the mechanical properties of basalt fiber-reinforced fully recycled concrete. The main conclusions are as follows:

(1) The proposed triple synergistic modification exhibits a notable enhancement in the mechanical properties of fully recycled concrete under the tested conditions. With the optimized parameters, the 28-day compressive strength and splitting tensile strength of the specimens reach 77.2 MPa and 9.45 MPa, representing increases of 56% and 129%, respectively, compared to the unmodified group. The relatively low data dispersion observed across modified specimens further indicates the stability of this modification system within the scope of this study.

(2) Each modified component is indispensable, and their synergistic effect depends on a specific optimal concentration range. The absence of any single component will lead to performance degradation; among these, the lack of nanofibers has the most significant impact on compressive strength, while the lack of dopamine exerts the greatest influence on tensile strength. Excessively high concentrations will cause problems such as excessively thick polymer films and fiber agglomeration, which will instead weaken the enhancement effect.

(3) Microscopic analysis reveals a three-level synergistic enhancement mechanism: pore filling, chemical bonding, and fiber bridging. Calcium carbonate fills the pores, dopamine enhances chemical bonding, and nanofibers inhibit the propagation of microcracks through bridging. These three components work together to reduce interfacial porosity, improve compactness, fundamentally optimize the interfacial structure, and thereby achieve a significant improvement in macroscopic properties.

(4) Although this study confirms the effectiveness of the triple synergistic modification, certain limitations remain: First, the modification process is relatively complex, affecting construction efficiency and economic viability. Second, systematic research on long-term durability (e.g., impermeability, carbonation resistance) is lacking. Third, the quantitative proportioning relationships among components require further universal validation.

(5) Future research can be pursued in the following directions: (1) Exploring simplified processes such as in situ polymerization to enhance engineering applicability; (2) Conducting systematic long-term durability tests to establish synergistic mechanisms between mechanical and durability performance; (3) Combining molecular dynamics simulations to reveal interfacial bonding mechanisms at the atomic scale, providing theoretical guidance for modifier design.

## Figures and Tables

**Table 1:** Mix Proportion Table.

Mixing Water (kg)	Fiber Length (mm)	Fiber Content (kg)	Cement (kg)	Coarse Aggregate (kg)	Fine Aggregate (kg)	Water-Reducing Agent (kg)	Micropowder (kg)
4.335	18	13.013	14.459	32.705	8.176	2.17 × 10^−4^	1.446

**Table 2:** Experimental Data.

Test Samples	Concentration of Calcium Ion Accelerating Solution (M)	Dopamine Solution (mg/mL)	Nanofiber Dispersion		Compressive Strength MPa	Splitting Tensile Strength MPa
			Mass Percentage of Nanofibers (%)	Mass Percentage of Anionic Surfactants (%)		
Example 1	0.1	5	0.05	1	77.2	9.45
Example 2	0.125	10	0.1	0.5	72.5	9.32
Example 3	0.0125	1	0.01	0.1	73.7	9.25
Example 4	0.125	10	0.1	0.1	73.5	9.04
Example 5	0.0125	1	0.1	1	75.5	9.13
Comparative Example 1	0.1	5	added directly as a filler		64.2	7.58
Comparative Example 2	/	5	0.05	1	61.3	6.59
Comparative Example 3	0.1	/	0.05	1	62.5	6.13
Comparative Example 4	0.1	5	/	/	59.8	6.36
Comparative Example 5	0.1	20	0.05	1	66.5	6.57
Comparative Example 6	0.1	5	0.2	1	65.1	6.25
Comparative Example 7	/	/	/	/	49.5	4.13

## Data Availability

The original contributions presented in this study are included in the article. Further inquiries can be directed to the corresponding author.
